# Accuracy deficits during robotic time-constrained reaching are related to altered prefrontal cortex activity in children with cerebral palsy

**DOI:** 10.1186/s12984-024-01502-x

**Published:** 2024-12-19

**Authors:** Owais A. Khan, Tarkeshwar Singh, Deborah A. Barany, Christopher M. Modlesky

**Affiliations:** 1 Department of Kinesiology, University of Georgia, 330 River Road, Athens, GA 30602 USA; 2 Department of Kinesiology & Penn State Neuroscience Institute, The Pennsylvania State University, University Park, PA 16802 USA; 3 Department of Interdisciplinary Biomedical Sciences, School of Medicine, University of Georgia, Athens, GA 30602 USA

**Keywords:** Cerebral palsy, Functional neuroimaging, Robotics, Functional near-infrared spectroscopy, Prefrontal cortex, Coordination, Motor planning, Reaching, Neuroplasticity, Motor control

## Abstract

**Background:**

The prefrontal cortex (PFC) is an important node for action planning in the frontoparietal reaching network but its role in reaching in children with cerebral palsy (CP) is unexplored. This case–control study combines a robotic task with functional near-infrared spectroscopy (fNIRS) to concurrently assess reaching accuracy and PFC activity during time-constrained, goal-directed reaching in children with CP. We hypothesized that reaching accuracy in children with CP would be lower than in typically developing children and would be related to PFC activity.

**Methods:**

Fourteen children with spastic CP (5-11 y; Manual Ability Classification System level I-II) and 14 age-, sex- and arm dominance-matched typically developing controls performed seated uniplanar reaches with a robotic arm (KINARM End-Point Lab) to hit visual targets projected onto a screen. Four blocks of 10 reaching trials each were performed for each arm. Time constraint (high, low) was varied across blocks by changing the time participants had to hit the target.

**Results:**

Children with CP displayed lower reaching accuracy compared to controls, with greater deficits observed in the non-preferred arm (*d* = 1.916, *p* < 0.001) than the preferred arm (*d* = 1.033, *p* = 0.011). Inter-limb differences in accuracy were observed only in children with CP (*d* = 0.839, *p* < 0.001). PFC activity differed across groups during preferred arm reaching, with PFC deactivation observed in children with CP under high time constraints compared to PFC activation in controls (*d* = 1.086, *p* = 0.006). Children with CP also exhibited lower PFC activity under high time constraint compared to low time constraint in the preferred arm (*d* = 0.702, *p* = 0.001). PFC activity was positively related to reaching accuracy across time constraints in both arms in children with CP, but not in controls.

**Conclusions:**

Contrasting patterns of PFC activity observed in children with CP compared to age- and sex-matched controls during a robotic reaching task lends support for the concurrent use of fNIRS and robotics to assess goal-directed reaching in CP.

*Trial Registration*: Data collected as part of a larger randomized controlled trial; https://clinicaltrials.gov/ct2/show/NCT03484078

## Background

Goal-directed reaching is a fundamental motor behavior that empowers children to build relationships, participate in play and sport, and achieve functional independence in activities of daily living. Successful reaching requires the rapid and continuous coordination of the visual, sensorimotor and attentional-perceptual neural systems [1]. This multi-system integration is especially challenging for children with neurodevelopmental disorders such as cerebral palsy (CP) [2, 3]. Affecting 1 in 323 children in the United States, CP is considered the most common cause of childhood-onset motor disability [4]. Children with CP exhibit central deficits in action planning [5, 6], visuospatial attention [7], eye-hand coordination [8, 9], and sensorimotor function [10, 11], all of which contribute to the impaired reaching behaviors observed in these children [12]. Understanding the neural correlates of these impairments is essential to developing targeted interventions to improve upper limb function in children with CP.

Robotic technologies have recently emerged as valid and reliable tools to assess the complex multi-system deficits observed in children with CP [13–20]. In addition to providing objective quantitative measures of movement dysfunction, robotic assessments can incorporate reliable, sensitive, and ecologically valid tasks that better translate to real-world performance [21] than subjective clinical assessments of manual abilities [22, 23]. Robotic technologies are safe, feasible, and well tolerated by young children with neurological impairments, and have been used to quantify upper limb sensorimotor [16–19], visuospatial [13], and executive functions [20] in children with perinatal stroke-induced CP. In line with previous reaching studies using motion capture techniques [24, 25], robot-derived reaching metrics revealed global deficits in reaching kinematics of children with arterial stroke-induced CP that were related to clinical metrics of manual ability [17]. The integration of neuroimaging and neurophysiological assessments alongside standardized robotic tasks can help researchers evaluate brain-behavior relationships in both neurotypical children and children with neurodevelopmental disorders, like CP. Prior work combining robotic assessment of reaching with neuroimaging revealed sensory deficits were associated with sensory tract development in children with perinatal stroke [14], highlighting the importance of assessing neurophysiological outcomes alongside robot-derived metrics to better understand the reaching deficits observed in CP.

Despite the promise of more sensitive assessments offered by integrated robotics-neuroimaging, the use of functional neuroimaging has been limited in children with CP due to their inability to stay still while remaining attentive and engaged throughout the assessment task. The recent emergence of portable, non-invasive neuroimaging technologies, like functional near-infrared spectroscopy (fNIRS), shows promise in overcoming this limitation [26]. Source optodes on fNIRS devices emit specific wavelengths of near-infrared light that pass through the skin and superficial tissue and are differentially absorbed by oxygenated and deoxygenated hemoglobin within the outer 5–10 mm strip of cortical grey matter underlying the surface optodes. Light waves re-emerge and are captured by detector optodes located at specific distances from the source optode. The light intensity differential indicates relative changes in hemoglobin concentrations that serve as indirect indices of neural activity in the underlying cortical regions spanning the source-detector channel [27]. Advantages of fNIRS include its portability, relative inexpensiveness, and robustness to motion artifacts compared to functional magnetic resonance imaging, and superior comfort, higher spatial resolution, and ease of application compared to electroencephalography [28]. These characteristics make fNIRS a promising tool for assessing task-induced cortical activity in adults [29] and children [26] with neurological impairments.

In contrast to these stated advantages, the fNIRS literature in CP is sparse. Most prior studies assessed sensorimotor cortex activation during simple single- and multi-joint movements [30–38]. An exploratory study reported increased activation in the prefrontal cortex (PFC) of two adults with CP during an upper limb aiming (ball-drop) task [39]. The PFC is an important node for action planning and prediction within the fronto-parietal neural network for goal-directed reaching [40, 41], priming the sensorimotor cortices for the expected sensorimotor inputs arising from novel and purposeful movements [42]. The PFC is considered a cortical hub for mediating visuospatial attention [43] and spatial localization [44] abilities essential for accurate targeted reaching [45]. While prior studies have evaluated PFC activation during reaching tasks in neurotypical adults [44, 46], the exploration of PFC activity in children with CP has been limited to motor planning and cognitive-motor dual-tasks [47–49]. The heightened PFC activity observed in children with CP during these dual-tasks was posited to represent neural resource re-allocation to compensate for their impaired posture and balance [48]. Interestingly, PFC activity diminished to levels comparable with typically developing controls following intensive training, suggesting PFC activity optimization may precede, accompany, or follow the improved functional abilities seen in children with CP post-intervention [49]. In contrast to the increased PFC activation observed in upper limb tasks, a recent fNIRS study reported lowered PFC activity in young children with CP during robot-assisted walking [50]. The increased PFC activity observed in these children following robot-assisted gait training was associated with improvements in functional mobility. These initial studies suggest that PFC activity may either serve as a potential neurophysiological marker for post-intervention functional gains, or more speculatively, be a relative therapeutic target in children with CP. Despite these promising preliminary observations, to the best of our knowledge, no study has determined PFC activity during goal-directed reaching in children with CP.

This study aimed to concurrently evaluate reaching accuracy and PFC activity in children with CP using a robotic goal-directed reaching task that incorporated distinct time constraints. We aimed to [1] determine if children with CP have deficits in reaching accuracy and a different level of task-evoked PFC activity compared to typically developing children, and [2] determine if reaching accuracy is related to PFC activity across differing time constraints. Based on the prior literature summarized above [13, 15, 17, 39, 44, 46–50], we hypothesized that [1] children with CP would exhibit lower reaching accuracy and higher PFC activity during the time-constrained reaching task than typically developing children, and [2] that PFC activation would be positively related to reaching accuracy in children with CP.

## Methods

### Participants

Children with spastic CP aged 5–11 years were recruited from schools, social media platforms, pediatric rehabilitation centers, Children’s Healthcare of Atlanta, and the Cerebral Palsy Foundation as part of a larger randomized controlled trial. Age- (± 1.5 years) and sex-matched typically developing children with no known neurological disorders and height and body mass between the 5th and 95th age- and sex-based percentiles were recruited as controls. Exclusion criteria for this study were [1] Manual Ability Classification System (MACS) level V (does not handle objects and has severely limited ability to perform simple actions) [2], cognitive impairments that limit the ability to follow simple verbal instructions [3], botulinum toxin injection in the past 6 months, and [4] significant visual acuity problems and/or upper limb musculoskeletal deformities that would preclude completion of testing. The Institutional Review Board at the University of Georgia approved this study. Prior to the study, written informed consent was obtained from the participants’ parent or legal guardian, and informed assent was obtained from the participant.

### Anthropometrics

Height and body mass were assessed while participants wore minimal clothing and were in bare feet. Height was measured using a stadiometer (Seca 217; Seca GmbH & Co. KG., Germany) to the nearest 0.1 cm, and body mass was measured using a digital scale (Detecto 6550, Cardinal Scale, MO, USA) to the nearest 0.1 kg. Body mass index (BMI) was calculated using height and body mass. Normative data published by the US Centers for Disease Control and Prevention [51] were used to determine age- and sex-based percentiles for body mass, height, and BMI. Hand preference was assessed using the Edinburgh Handedness Inventory-Short Form [52] to confirm right-arm preference for all participants.

### Clinical measures

Manual ability was classified by the parent/guardian using the MACS [53]. Gross motor function was assessed using the Gross Motor Function Classification System (GMFCS) [54]. Muscle tone was assessed using the Hypertonia Assessment Tool (HAT) [55].

### Functional neuroimaging (fNIRS) setup

**Fig. 1:**
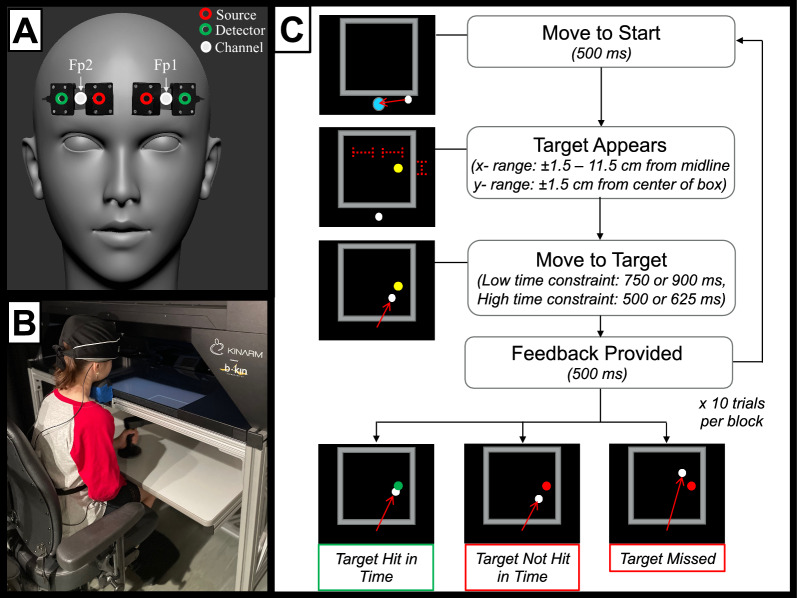
Study methods. **A** Functional near-infrared spectroscopy device placement over the regions of interest in the left PFC (Fp1) and right PFC (Fp2), based on the International 10/20 system. **B** Experimental setup with the participant positioned within the KINARM robotic device. **C** Trial protocol for the robotic time-constrained reaching task

Two lightweight, portable (Bluetooth-enabled) fNIRS devices (Portalite, Artinis Medical Systems, The Netherlands) were secured to the participant’s forehead, as depicted in Fig. 1A. The device has 3 optodes (sources) set at fixed distances of 3, 3.5, and 4 cm from a single fixed ‘detector’, yielding three spatially overlapping source-detector channels. Near-infrared light at two wavelengths (850 nm, 760 nm) was emitted from the source optodes, with devices centered over the Fp1 and Fp2 locations of the International 10–20 system to cover the bilateral prefrontal cortices. Data from the 3 cm channel were used for further analysis, in line with manufacturer recommendations and previous studies that used the same device in typically developing children [56]. To minimize interference from ambient light, a black felt cloth was placed over the fNIRS devices and secured with a black cap. Data were sampled at 50 Hz and visualized in real-time with the manufacturer’s data recording software (Oxysoft v3.2.64 × 64, Artinis).

### Experimental setup

The general experimental setup was adapted for children from a previous study [57] and is illustrated in Fig. 1B. Briefly, participants sat on a height-adjustable chair with their feet supported on an adjustable footrest. They used one hand to grasp the handle of a robotic device (KINARM End-Point Laboratory, Ontario, Canada). Handle movement in the horizontal plane controlled the position of a cursor (white circle, 1 cm diameter) on a screen comprised of a semi-transparent mirror. All visual stimuli were projected onto this screen at 60 Hz or 120 Hz from a monitor at the top of the workspace to ensure the cursor and visual stimuli were on the same horizontal plane. Participants could track the location of the cursor on-screen during the reaching movements, but they could not see the physical movements of the arm during the movements (i.e., no direct visual feedback) as the hand was occluded from direct sight by the screen. Handle position and velocity of movement were recorded at 1000 Hz. To minimize head motion, participants were instructed to stabilize their chin on a fixed chinrest with the crown of their head against the KINARM workspace boundary, and to fix their gaze on the cursor and target for the entire task.

The trial protocol is summarized in Fig. 1C. Briefly, each trial began with the participants moving the cursor to a start position (2 cm blue circle) in the midline of the visual display (x = 0) directly below the workspace (34 by 34 cm box centered on the midline). If the cursor position was maintained for 500 ms, the start position disappeared, and the target (yellow circle, 1 cm diameter) appeared onscreen. The target location on each trial was randomized, with targets appearing on the left or right side of the workspace at equal probability. The x-position of the target was constrained within a uniform distribution around a mean position ± 6.5 cm from the workspace mid-line (range ± 5 cm). The y-position of the target was constrained within a uniform distribution around the center of the workspace (mean position 19 cm from the start position, range ± 3 cm). Participants were instructed to move the cursor to hit the target as rapidly and accurately as possible for every trial. They were not required to stop at the target location. A “hit” was recorded when the cursor first overlapped with the target. Performance feedback was provided for 500 ms once the object was hit or the maximum trial duration was reached. If the target was hit within the block-specific time constraint, it would turn green (“hit”); if the target was missed or hit after the time constraint period, it would turn red (“miss”). A 2000 ms delay was incorporated between successive trials.

Targets remained onscreen for a maximum of 500 ms or 625 ms for the high time constraint blocks, and 750 ms or 900 ms for the low time constraint blocks. Before and after each block, participants were instructed to let go of the handle, and look at the cursor onscreen for 25–30 s (randomized) to allow PFC hemodynamics to return to baseline levels. The preferred (right) arm was always assessed first, with 4 blocks performed with each arm and the block order randomized within each arm. Each block comprised 10 trials (80 trials total), with a time constraint defined and maintained for all trials within a block.

### fNIRS data processing

All fNIRS data were preprocessed in MATLAB^®^ (MathWorks, Natick, MA, USA) using the HOMER3 (v1.28.1) software package [58]. Current best practices for fNIRS data processing were followed [59]. First, frequency distributions of all channels and both wavelengths were visually inspected with power spectral density graphs to detect the characteristic hemodynamic ‘pulse’ at 1–2 Hz that signifies good signal quality. Raw light intensity signals were subsequently converted into changes in optical density (*Intensity2OD* function) and submitted through a low-pass filter (*BandpassFilt* function with parameters *hpf* = 0 Hz, *lpf* = 0.09 Hz) to account for components from systemic physiology (e.g., cardiac pulsations, respiratory cycles). The filtered time series data were then corrected for motion artifacts using the hybrid spline interpolation-Savitzky Golay procedure (*MotionCorrectSplineSG* function with parameters *p* = 0.99, *FrameSize* = 10 s) chosen for its efficiency and effectiveness in accounting for both baseline shifts (via spline interpolation) and sharp motion artifacts (via robust, locally weighted smoothing through the Savitzky-Golay filter) [60]. Motion-corrected optical density data were converted into relative changes in hemoglobin concentration using the modified Beer-Lambert law (*OD2Conc* function with parameters *ppf* = 1, 1). Data were then baseline corrected by subtracting the mean of the signal from the 2 s preceding each trial and block averages for changes in hemoglobin concentration were calculated for each block (*BlockAvg* function with parameters *trange* = −2 to 45 s). Baseline-corrected time-series block averages were exported and mean values were calculated for a time period spanning 0 to 45 s post-trial onset. Mean changes in concentration of oxyhemoglobin (∂HbO) were used as markers of brain activation as oxyhemoglobin has previously been shown to be a more sensitive indicator of neural activity in the PFC than deoxyhemoglobin [61, 62] and has been the chromophore of choice to assess PFC activity in children with CP [47–49].

### Statistical analysis

Statistical analyses were conducted in RStudio (v2022.07.2, R Core Team 2022) and SPSS (v27.0.1, IBM Corp., Armonk, NY). Data were examined for normality by assessing skewness and kurtosis values and using the Shapiro-Wilk test. Group differences in physical characteristics were assessed using independent t-tests for normally distributed data, and Mann-Whitney *U* tests for non-normally distributed data. One-sample t-tests were used to assess if height, body mass, and BMI percentiles differed from 50th percentiles for age- and sex-based norms. Linear mixed-effect models were used for the performance outcome (reaching accuracy) with fixed effects of group (CP vs. controls), arm (preferred vs. non-preferred), time constraint (high vs. low) nested within subject (random effect). Linear mixed-effect models were also used for the fNIRS outcome (Change in HbO) with the addition of PFC hemisphere (ipsilateral vs. contralateral) as a fixed effect, as these statistical models can account for the inherently nested structure of fNIRS data [63] and the correlations arising from repeated measurements [64]. Pairwise comparisons were conducted for significant interactions and main effects. Alpha was set at 0.05 a priori and a multiple comparison correction was conducted using the Benjamini–Hochberg procedure [65]. The relationship between reaching accuracy and PFC activity was assessed using Spearman rank correlation (*r*_*s*_). Effect sizes were determined using Cohen’s d (*d*), with 0.2, 0.5 and 0.8 representing small, medium, and large effect sizes, respectively [66]. Data are presented as mean ± SD in the text and tables and as mean ± SE in figures, unless specified otherwise.

## Results

### Participant characteristics

Fourteen children with spastic CP and fourteen age- and sex-matched typically developing control children met the inclusion criteria for the study. Participant characteristics are presented in Table 1. No between-group differences were detected for any physical characteristic (all *p* > 0.05) and percentiles for height, body mass, and BMI were not different from the 50th age- and sex-based percentiles for either group (all *p* > 0.05).

**Table 1:** Physical characteristics of children with cerebral palsy (CP) and typically developing control children (Con)

	CP (n = 14)	Con (n = 14)	*d*	*p*
Age (years)	8.7 ± 1.7	8.6 ± 1.9	0.094	0.815
Sex (male/female)	9/5	9/5	–	–
Height (m)	1.30 ± 0.13	1.32 ± 0.11	0.167	0.927
Height (%)	38 ± 34	59 ± 25	0.677	0.073
Body mass (kg)	30.6 ± 7.7	29.7 ± 9.0	0.110	0.520
Body mass (%)	58 ± 30	55 ± 28	0.074	0.846
BMI	18.0 ± 3.4	16.7 ± 2.4	0.469	0.233
BMI (%)	60 ± 34	54 ± 29	0.195	0.370
Arm dominance (right/left)	14/0	14/0	–	–
CP diagnosis (unilateral/bilateral)	3/11	–	–	–
GMFCS level (I/II)	11/3	–	–	–
MACS level (I/II)	4/10	–	–	–
HAT (right arm)	11^a^/2^b^/0^c^/1^d^	14^a^/0^b^/0^c^/0^d^	–	–
HAT (left arm)	8^a^/5^b^/0^c^/1^d^	14^a^/0^b^/0^c^/0^d^	–	–

All values are mean ± SD. % represents percentiles for height, body mass, and body mass index (BMI), none of which were significantly different from the age- and sex-based 50th percentiles. Gross motor function indicated by Gross Motor Function Classification System (GMFCS) rating. Manual ability indicated by Manual Ability Classification System (MACS) rating. Muscle tone abnormality indicated by Hypertonia Assessment Tool (HAT) rating as ^a^normal tone, ^b^spasticity, ^c^dystonia, ^d^mixed tone (spasticity and dystonia)

### Reaching accuracy

**Fig. 2:**
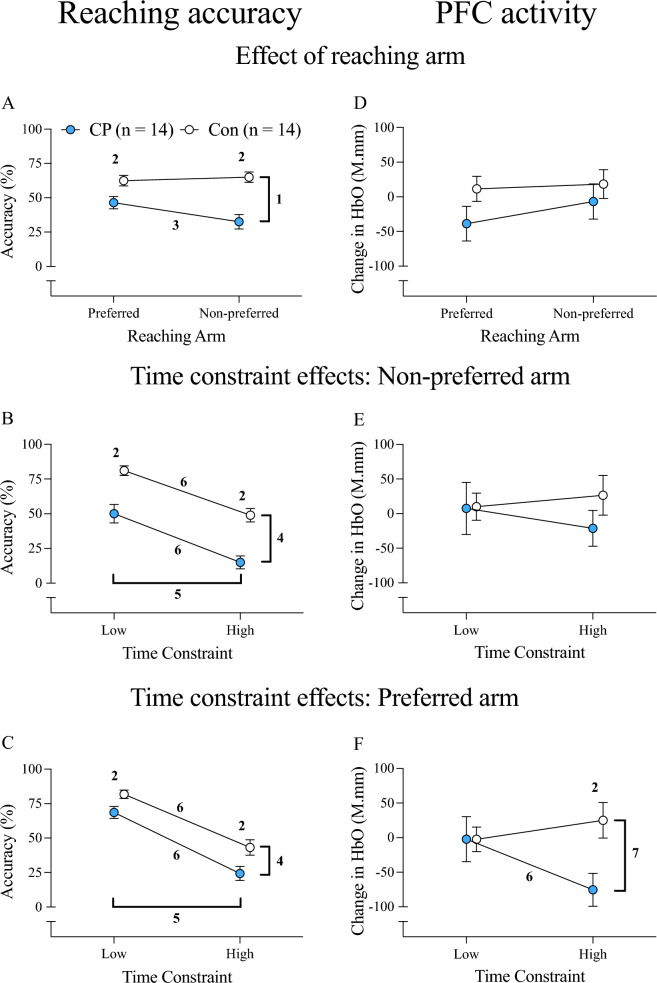
**Reaching accuracy and overall PFC activity (averaged across hemispheres) for time-constrained reaching.** Results are presented for each arm (**A** and **D**), and for each time constraint during non-preferred arm (**B** and **E**) and preferred arm reaching (**C** and **F**). Values are mean ± SE; ^1^group x arm interaction; ^2^group difference; ^3^arm difference; ^4^main effect of group; ^5^main effect of time constraint; ^6^time constraint difference; ^7^group x time constraint interaction; *HbO* oxyhemoglobin, *CP* Cerebral palsy, *Con* Typically developing controls, *r*_*s*_ Spearman rho

The effect of group, reaching arm, and time constraint on reaching accuracy is illustrated in Fig. 2. A significant group-by-arm interaction was observed for reaching accuracy (*p* < 0.001; Fig. 2A). Post-hoc tests indicated that children with CP were less accurate than controls with both arms, but the accuracy deficits were twice as large with the non-preferred arm (mean difference = −32 ± 6%, *d* = 1.916, *p* < 0.001) than the preferred arm (mean difference = −16 ± 6%, *d* = 1.033, *p* = 0.011). Inter-limb differences in accuracy were observed in children with CP, with lower accuracy noted with the non-preferred arm (mean difference = −14 ± 17%, *d* = 0.839, *p* = 0.008). In contrast, no inter-limb differences in accuracy were observed in controls (*d* = 0.170, *p* > 0.05). A significant main effect of time constraint was observed with both the non-preferred arm (*p* = 0.012; Fig. 2B) and the preferred arm (*p* < 0.001; Fig. 2C).

### PFC activity

Effects of group, reaching arm, and time constraint on overall PFC activity are also visualized in Fig. 2. There were no group or arm effects on overall PFC activity (both *p* > 0.05; Fig. 2D). There were no group or time constraint effects on PFC activity when reaching with the non-preferred arm (both *p* > 0.05; Fig. 2E). A significant group-by-time constraint interaction was noted for overall PFC activity only during preferred arm reaching (*p* = 0.001; Fig. 2F). Post-hoc tests indicated that children with CP had lower PFC activity than controls when reaching under high time constraints (mean difference = −100.5 ± 35.4 M.mm, *d* = 1.086, *p* = 0.008), with no group differences observed under low time constraints (*d* = 0.003, *p* = 0.994). Children with CP also exhibited lower PFC activity under high time constraints compared to low time constraint reaching (*d* = 0.702, *p* = 0.021). A within-group difference was not observed in controls (*d* = 0.295, *p* = 0.290).

### Relationship between overall PFC activity and reaching accuracy

**Fig. 3:**
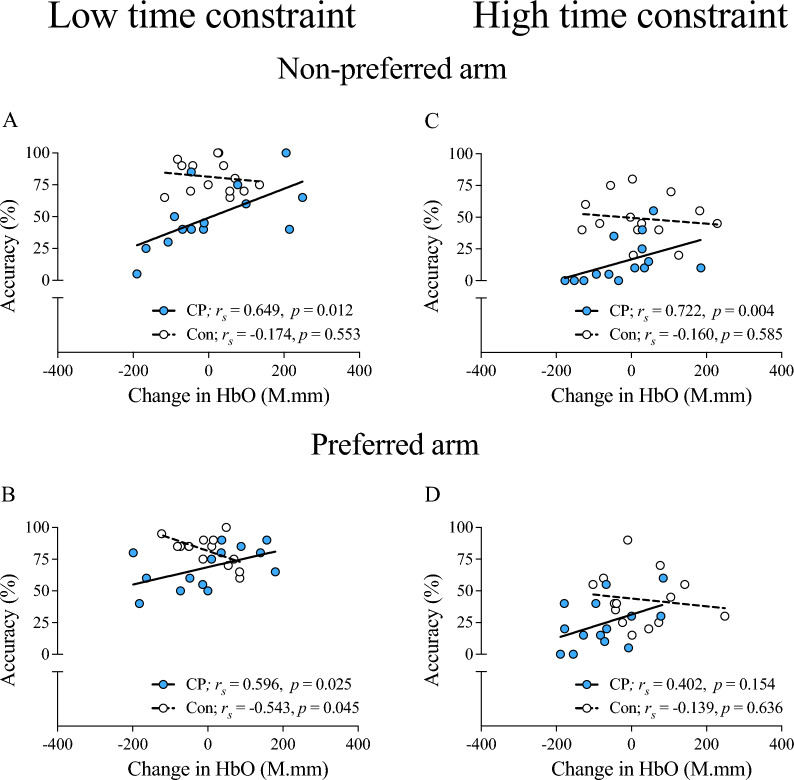
**Relationship between overall PFC activity and reaching accuracy.** Relationships of overall PFC activity with reaching accuracy for low time constraint (**A**, **B**) and high time constraint (**C**, **D**) reaching with the non-preferred arm (**A**, **C**) and the preferred arm (**B**, **D**) are illustrated. *PFC* Prefrontal cortex, *HbO* oxyhemoglobin, *CP* Cerebral palsy, *Con* Typically developing controls, *r*_*s*_ Spearman rho

Relationships between reaching accuracy and average PFC activity are presented in Fig. 3. For reaching done under low time constraints, positive relationships were observed only in the children with CP, with moderate-to-strong relationships noted for the non-preferred arm and the preferred arm (both *p* < 0.05; Fig. 3A, B). For high time constraint reaching, a strong positive relationship was observed in children with CP with the non-preferred arm (*p* = 0.004; Fig. 3C), but a significant relationship was not detected with the preferred arm (*p* > 0.05; Fig. 3D). Interestingly, these relationships consistently trended in the opposite (i.e., negative) direction for controls across time constraints in both arms, with a significant negative relationship noted only under low time constraints during preferred arm reaching (*p* = 0.045; Fig. 3B).

### Relationship between ipsilateral PFC activity and reaching accuracy

**Fig. 4:**
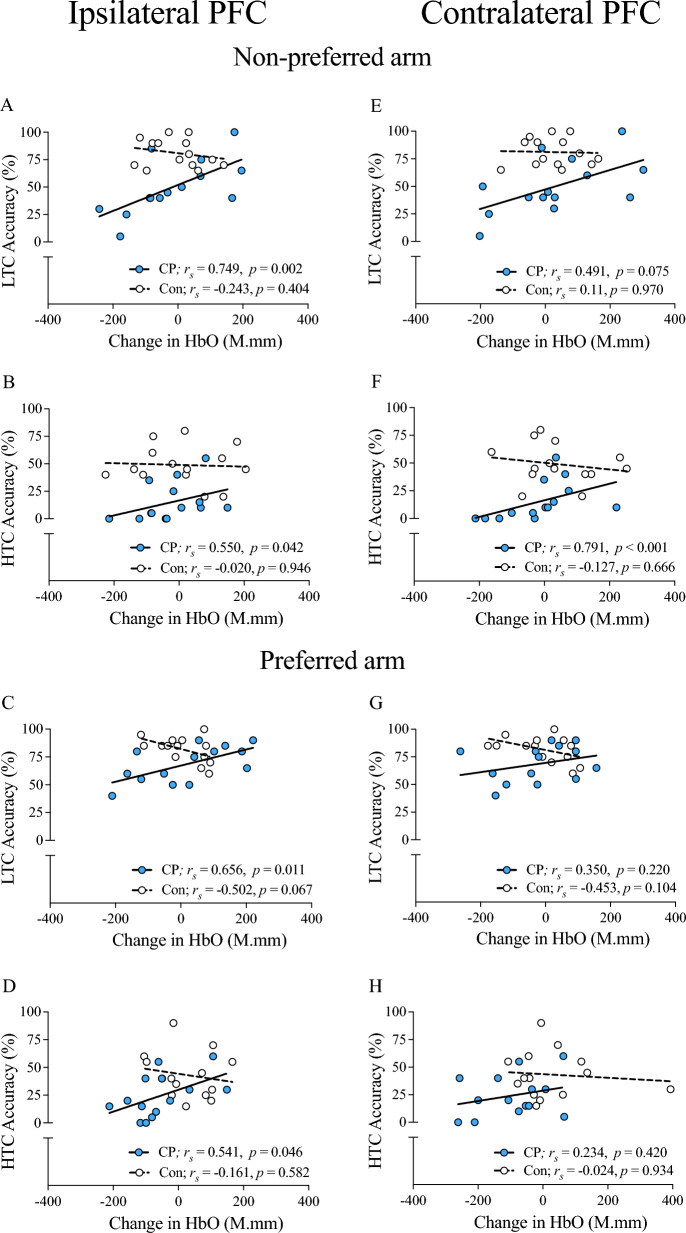
**Relationships between hemispheric PFC activity and reaching accuracy. **Relationships of ipsilateral PFC (**A**–**D**) and contralateral PFC (**E**–**H**) activity with reaching accuracy for preferred arm (**C**, **D**, **G**, **H**) and non-preferred arm (**A**, **B**, **E**, **F**) reaching are illustrated for the low and high time constraint conditions. *PFC* Prefrontal cortex, *LTC* Low time constraint, *HTC* High time constraint, *HbO* oxyhemoglobin, *CP* Cerebral palsy, *Con* Typically developing controls, *r*_*s*_ Spearman rho

Relationships between ipsilateral PFC activity and reaching accuracy are depicted in Fig. 4. Moderate-to-Strong positive relationships were detected in children with CP when reaching under low and high time constraints, with both the non-preferred arm and the preferred arm (all *p* < 0.05; Fig. 4A-D). No significant relationships were observed in controls (all *p* > 0.05).

### Relationship between contralateral PFC activity and reaching accuracy

Figure 4 also depicts relationships between contralateral PFC activity and reaching accuracy. A strong positive relationship was noted in children with CP when reaching under high time constraints with the non-preferred arm (*p* < 0.001; Fig. 4F), but not with the preferred arm (*p* > 0.05; Fig. 4H). Similarly, positive but non-significant trends were noted for children with CP with low time constraint reaching with either arm (both *p* > 0.05; Fig. 4E, G). None of these relationships were significant for controls (all* p* > 0.05).

## Discussion

In this study, we examined reaching accuracy and PFC activation during a time-constrained reaching task across both upper limbs in children with CP. As hypothesized, children with CP exhibited deficits in visuomotor accuracy compared to age-, sex-, and arm dominance-matched typically developing controls, with deficits accentuated when reaching with the non-preferred arm. Interestingly, imposing high time constraints adversely impacted reaching accuracy to a similar degree in both children with CP and typically developing controls, regardless of the arm used for reaching. Group differences in overall PFC activity were noted only when reaching with the preferred arm. Contrary to expectation, children with CP displayed PFC deactivation under high time constraints compared to low time constraints, while typically developing controls showed the opposite but non-significant trend of increased PFC activation. Moderate-to-strong positive relationships between overall PFC activity and reaching accuracy were observed in children with CP, both when reaching under low time constraints (for each arm) and under high time constraints (for non-preferred arm only). Correlation analyses suggested distinct roles of each PFC hemisphere during time-constrained reaching in children with CP. Specifically, there were moderate-to-strong positive relationships between the ipsilateral PFC activity and accuracy when reaching under low time constraints for both arms. In contrast, contralateral PFC activity was positively related to accuracy only when reaching under high time constraints with the non-preferred arm. No significant relationship between PFC activity and accuracy was observed with controls. Overall, these novel findings suggest that reaching performance and PFC activity in children with CP depends on both imposed time constraints and the arm used for reaching.

### Reaching accuracy

The pronounced inter-limb differences in reaching accuracy observed in children with CP is in line with previous studies [67] that report similar deficits in children with CP during time-constrained reaching with the non-preferred arm. Children with spastic CP have recognized motor control deficits that hinder reaching performance, with slower, less flexible, and more fragmented movements observed in the non-preferred arm [67, 68]. Time-constrained reaching places increased demands on the neuromuscular system that contribute to markedly different movement strategies for each arm during reaching in CP [69]. As in previous studies, we did not observe inter-limb differences in reaching accuracy in controls, indicating that accuracy deficits were specific to children with CP and not due to general hand dominance or lateralization effects [67]. We also noted that children with CP had lower reaching accuracy than controls, even when reaching with the preferred arm and under low time constraints. These observations are in line with the growing body of evidence detailing sensorimotor deficits in the less-impaired (preferred) arm of children with CP, with impaired domains including manual function [70], motor planning, and most pertinent to this study, visually-guided reaching and hitting performance [13, 17, 67, 71].

In contrast to our hypothesis, we observed that time constraints did not differentially affect reaching accuracy across groups, in either the preferred or the non-preferred arm. This finding could be due to the relatively lower emphasis placed on spatial accuracy compared to movement speed in the current study. Our time-constrained task emphasized movement speed (ability to move faster to reach the target within shorter time duration) over endpoint variability (i.e., ability to maintain cursor position at the target) as participants were not explicitly instructed to stop at the target location. Consequently, the movements lacked the deceleration needed to bring the arm to a fixed end-point, thus minimizing the computational burden placed on the effector system to bring the limb to a stable end-position [72]. This is a key consideration as preliminary work indicates that children with CP do not adhere to the typical speed-accuracy trade-off (i.e., Fitts’ law) commonly observed in healthy adults and typically developing children [73]. Children with CP perform better at tasks that emphasize movement speed over end-point accuracy [74]. Another factor could be that the outcome measure for reaching accuracy (i.e., dichotomous ‘hit/miss’ accuracy rate) for this pilot study was not sensitive enough to parse out finer between-group differences in reaching performance across time constraints. Processing of the richly detailed kinematic data provided by the KINARM robot may provide more sensitive outcomes of reaching performance (e.g., movement path variability, initial direction error) [17, 67], but these analyses were beyond the scope of this study. Finally, the imposed time constraints may not have been sufficiently challenging; however, similar declines in reaching accuracy observed at the high time constraint condition in both groups point to the adequacy of the selected time constraints in assessing reaching performance.

### Prefrontal cortex activity

Our results indicated that children with CP exhibited lower overall PFC activity during preferred arm reaching under high time constraints, relative to both controls and reaching performed under low time constraints. A similar deactivation pattern, also specific to CP, was observed during non-preferred arm reaching under high time constraints, though this did not reach statistical significance. These novel results contradict our stated hypotheses of increased PFC activity in children with CP under high time constraints. Surkar and colleagues reported increased PFC activity in children with hemiplegic CP relative to typically developing controls during a sequential shape-matching cognitive task performed in isolation [47, 49] and under conditions of increased postural demands (cognitive-motor dual-task) [48]. One explanation for the difference in PFC activation patterns observed in our study is that our time-constrained reaching task requires greater response speed and high movement amplitudes, placing significantly greater demands on the visuo- and sensory-motor systems. Children with CP require greater neural resources to produce similar levels of motor output for both automated behaviors like walking [35, 75] and purposeful movements like goal-directed reaching. Impaired cortical processing of visuospatial [9, 76], proprioceptive [77], and sensorimotor information [78] in children with CP may also contribute to their impaired upper limb function [77]. With abnormally high sensorimotor cortex activity reported in CP during simple uni-joint movements [36, 37], performing more challenging tasks like time-constrained reaching may require neural resource re-allocation [79] away from cognitive-centered prefrontal cortices toward caudal sensorimotor and multisensory association cortices in children with CP [80].

Recent work from neuroeconomics attempts to model this complex ‘constrained resource allocation’ [79]. Alonso et al. [79] highlight the role of the PFC as an important node in the proposed central executive system that regulates neural resource allocation. This higher-order function of the PFC supports its critical role in mediating executive functions, such as action planning, spatial attention, working memory, and error monitoring [81]. In contrast to these critical functions, we observed a decrease in neural resource allocation to the PFC (i.e., deactivation) in children with CP when reaching was performed under high time constraints. Prior work in children with unilateral CP [34] and adults with stroke [82] may help explain this unexpected observation of PFC deactivation in CP. Increased sensorimotor cortical activation observed in the lesioned hemisphere of individuals with stroke during manual task performance was positively related to force production [82]. Neuroimaging studies in children with CP found brain activation to be excessive [83] and widespread [84], revealing the tendency of the damaged CNS to divert its limited neural resources to functionally important yet neurologically impaired brain areas. Notably, the magnitude of the proposed compensatory neural resource re-allocation and its impact on task performance appears co-dependent on task demands and environmental context in CP [85]. Abnormally increased activity observed in the contralateral sensorimotor cortex in children with unilateral CP was related to less discrete muscle activation patterns and accompanied by inefficient movement trajectories during complex upper limb tasks like simulated pouring and asymmetric squeezing [34]. Specific to the current study, similar patterns of lowered overall PFC activity were observed in children during high time constraint reaching with both the preferred and non-preferred arms. Though group differences in overall PFC activity were only statistically significant in the preferred arm, strong associations between overall PFC activity and reaching accuracy were observed in children with CP during non-preferred arm reaching under high time constraints. The lower magnitude of PFC de-activation displayed by children with CP during the latter condition may also indicate attenuated resource reallocation through greater attentional focus, mental effort, or impulse control, or that other factors, such as altered movement kinematics, may influence PFC activation in CP. Taken together, these observations complement previous reports suggesting PFC activation is a potentially sensitive marker of task performance in children with CP [49], especially when movement challenge [86] and task demands [50] are increased, raising the possibility that PFC activation levels may serve as therapeutic targets for future interventions in this population.

### Relationship between PFC activity and reaching accuracy

The impact of the proposed neural resource re-allocation on reaching accuracy may be reflected by the correlational analyses results. In agreement with our initial hypothesis, we observed a consistent positive relationship between PFC activity and reaching accuracy in children with CP. This observation implicates the PFC as a potentially important contributor to reaching accuracy in children with CP; the corollary is that the observed PFC *de*-activation observed in children with CP may be a pathological, counterproductive phenomenon which adversely impacts reaching performance. The distinct PFC activity patterns observed in children with CP may serve as neurophysiological markers of functional performance. More speculatively, changes in PFC activity following intervention may accompany, precede, or follow functional gains in motor performance. Prior fNIRS work reported altered PFC activity in children with CP at the pre-intervention baseline was attenuated to levels comparable to typically developing controls following intensive upper limb therapy [49]. Another study that assessed fNIRS-derived measures of cortical activity during robotic-assisted gait training reported increased PFC activity in children with moderate-to-severe spastic CP at the conclusion of 12 sessions of training [50]. In both studies, the proposed ‘normalization’ of PFC activity was accompanied by functional improvements observed following each intervention. In contrast to the positive associations noted in CP, we observed negative trends signifying inverse relationships between overall PFC activity and reaching accuracy in controls, with a significant negative association noted only during preferred arm reaching under low time constraints. Notably, PFC activity is sensitive to task familiarization [44] and practice-based motor learning [87] in healthy adults, and could potentially indicate motor skill in neurotypical adults. Novel fNIRS work on surgical trainees revealed PFC activity patterns elicited during bimanual simulated surgeries could accurately distinguish between and classify surgical trainees (highest PFC activity), novice surgeons (low PFC activity), and experienced surgeons (lowest PFC activity) [88], suggesting PFC activity indicates skill acquisition and learning during complex upper limb tasks in neurotypical individuals [89]. These findings highlight the need for further research on task-induced PFC activation in both typically developing and neurologically impaired children to better understand the functional relevance of the distinct PFC activation patterns observed in children with CP.

The imposition of time constraints revealed novel nuances in the PFC activity-reaching accuracy relationship in children with CP that are hemisphere-specific. Specifically, the ipsilateral PFC showed a consistent positive relationship with reaching accuracy in both arms, with stronger associations observed under low time constraints. In contrast, the contralateral PFC showed a strong positive relationship with reaching accuracy only during non-preferred arm reaching performed under high time constraints. These results are in line with those of an exploratory pilot fNIRS study that observed PFC activity was lateralized to the ipsilateral PFC in two adults with CP during a ball throwing motor task compared to bilateral dominance seen in the healthy adult controls [39]. While no statistical conclusions could be drawn, the authors speculated that the PFC’s functional response to a visuomotor upper limb task may differ in CP compared to healthy individuals. Our novel observations support this exploratory hypothesis and shed light on a previously unexplored aspect of the cortical regulation of reaching in CP. In summary, these findings illustrate the complexity of the cognitive-visual-sensorimotor system interaction that underlies successful goal-directed reaching in children with CP.

The novel observations reported above are supported by several strengths in our study design. Our study sample, while modest in size, was tightly controlled, with only right-arm dominant participants in both the age- and sex-matched groups that did not differ in any commonly measured physical characteristics. We attempted to minimize the heterogeneity of the CP group by restricting enrollment to participants who were within a reasonably narrow age range (i.e., 5-11 years), exhibited clinically detectable spasticity, were independent ambulators (i.e., GMFCS levels I and II), and had mild impairments in manual abilities (i.e., MACS levels I and II). Motions artifacts commonly reported in work using fNIRS with neurological populations [90] were minimized during data collection by stabilizing the head within the KINARM frame and by using a chinrest, with additional automated motion artifact correction performed during data processing. Our reaching task was methodologically rigorous, with target location spatially randomized in a uniform distribution in both the x- and y- direction to minimize task predictability and increase trial-by-trial variability to prevent the decline in PFC activity observed across similar trials [44].

This work also has limitations. First, fNIRS technology is limited to the collection and interpretation of neurophysiological data from the outermost layer of the cerebral cortex; thus, we could not image sub-cortical regions like the inter-connected basal nuclei and cerebellar networks that also play an important role in movement planning and coordination [91]. The low spatial resolution of the fNIRS device we used (i.e., Portalite, Artinis) also precluded imaging of other areas within the PFC and other cortical regions of interest like the sensorimotor and parietal cortices. While restricted in its spatial resolution, the fNIRS device has previously been used to assess PFC hemodynamics in school-aged children [56, 92]. Second, the fNIRS device used also lacks the integration of short-separation channels that could account for hemodynamic changes arising from systemic physiology (i.e., blood pressure, respiration, heart rate changes, etc.). Though we cannot exclude the possibility of fNIRS signal contamination from these variables, recommendations for best practices in fNIRS processing were followed to minimize these contaminants [59]. Third, the modest sample size did not allow us to conduct further sub-group analyses (e.g., unilateral versus bilateral CP) and restricts the generalizability of our results. Despite this limitation, statistically significant group differences in reaching accuracy and PFC activity were noted, with significant positive relationships between reaching accuracy and PFC activity also detected in the children with CP. Finally, we did not collect data on eye movements, perceptual impairments, or proprioception that could shed further light on the underlying bases of the observed accuracy deficits during reaching in children with CP. However, previous work examining the relationship between proprioception and robotic reaching performance in children with hemiplegic CP showed a surprising dissociation of proprioceptive impairments from reaching performance, highlighting that perinatal stroke may differentially affect the sensory and motor systems [18]. We recognize that more detailed kinematic analyses could provide greater insights into the limb- and time constraint-specific deficits observed in children with CP, with the slower, jerkier, more variable, and less accurate reaching movements displayed by children with spastic CP [67, 93, 94] all potentially contributing to the observed accuracy deficits reported in this study. Detailed multivariate analyses of reaching kinematics [95] should be the focus of future studies to assess the role of specific kinematic parameters such as movement trajectory characteristics and velocity profiles on reaching performance across time constraints in children with CP.

## Conclusions

Our study is the first to reveal distinct PFC activity patterns in children with spastic CP during robotic time-constrained reaching. Specifically, contrasting patterns of PFC activity were noted across time constraints in children with CP (i.e., PFC deactivation) compared to age-, sex-, and arm dominance-matched typically developing controls (i.e., PFC activation). Correlation analyses revealed moderate-to-strong relationships between PFC activity and reaching accuracy across time constraints, with PFC activation patterns indicating possibly distinct roles of each hemisphere during time-constrained reaching in children with CP.

Our results support the feasibility of using fNIRS in conjunction with robotic technologies to simultaneously assess the cortical correlates and performance metrics of goal-directed upper limb movements in children with CP.

## Data Availability

Data used and/or analyzed for the current study will be made available from the corresponding author upon reasonable request.

## Abbreviations

CP: Cerebral palsy
fNIRS: Functional near-infrared spectroscopy
PFC: Prefrontal cortex
Con: Typically developing controls
BMI: Body mass index
MACS: Manual Ability Classification System
GMFCS: Gross Motor Function Classification System
HAT: Hypertonia Assessment Tool
HbO: Oxyhemoglobin
LTC: Low time constraint
HTC: High time constraint
